# EHMTI-0305. Psychological assessment of patients with chronic refractory headache: a consecutive case series

**DOI:** 10.1186/1129-2377-15-S1-D52

**Published:** 2014-09-18

**Authors:** F Galli, C Tassorelli, N Ghiotto, E Guaschino, M Allena, F Antonaci, G Sandrini, G Nappi, G Sances

**Affiliations:** 1 Headache Science Center, C. Mondino National Neurological Institute, Pavia, Italy

## Background

Refractory headaches (RH) represent a difficult challenge, even for the most skilled practitioners.

## Aim

In-depth psychological and psychopathological evaluation of patients with RH.

## Methods

We enrolled 51 consecutive patients (age range 24-65 years) with a chronic headache who satisfied 1 or more of the following conditions: 1) failure of at least 2 prophylactic treatments; 2) medication overuse non-responsive to at least 2 properly conducted protocols of detoxification; 3) medication overuse relapsed at least twice after successful detoxification. Patients were evaluated by a clinical psychologist by the means of SCID-I (for psychiatric disorders), SWAP-200 (for personality) and investigation of life/traumatic events.

## Results

Ninety-six-percent of the patients showed at least one psychiatric disorders of the anxiety-depression spectrum: 65% at least one anxiety-disorder, 63% at least one mood-disorder and 50% one of somatoform-disorders with 67.65% showing more than one disorder. No patient showed Obsessive-Compulsive disorder or Headache Attributed to Psychiatric disorders (ICHD-III-β). As regards personality clinical characteristics, a relevant portion of subjects showed obsessive personality prototype (43.5%), dysphoric high-functioning personality prototypes was detected in 23% of subjects, while clinical traits of Histrionic and Avoidant personality clinical prototypes were present in 20.6% and 35%, respectively. None of the subjects showed Schizoid, Anti-Social, Emotional Dysregulated, Dependent or Hostile personality prototypes. Forty-seven percent showed some kind of infantile trauma/abuse and 88% referred at least one stressful event after headache onset.

## Conclusion

Psychological and psychopathological factors are of critical importance in RH, and should be taken into consideration for both diagnostic and therapeutic purposes.

No conflict of interest.

